# Supplementary figures and images for: Prolonged Intrinsic Neural Timescales Dissociate from Phase Coherence in Schizophrenia
Source: Brain Sci. 2023 Apr 21;13(4):695. doi: 10.3390/brainsci13040695 (PMC10137081; doi:10.3390/brainsci13040695)

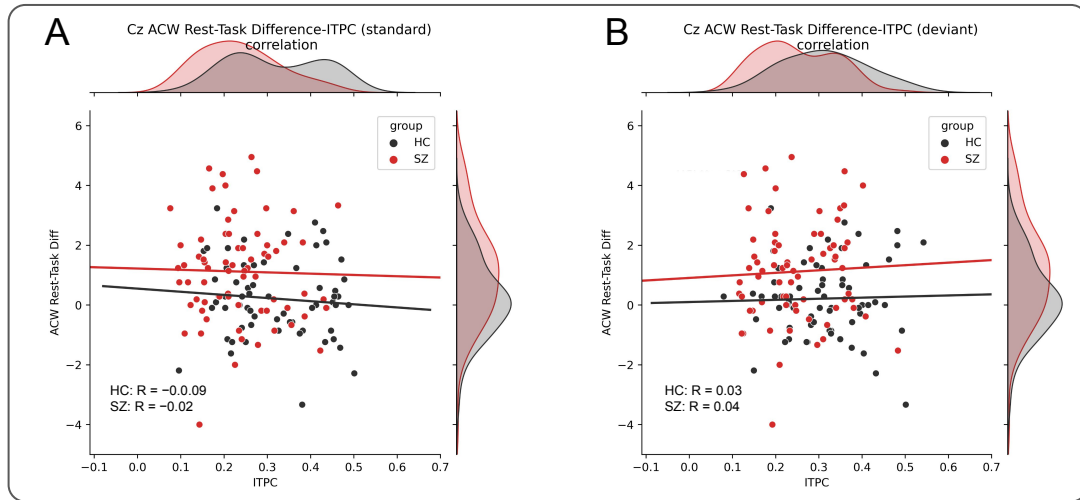

Supplement: Supplementary file 1 [file brainsci-13-00695-s001.zip › brainsci-2342908-supplementary.pdf]
